# Data reproducibility of pace strategy in a laboratory test run

**DOI:** 10.1016/j.dib.2016.03.057

**Published:** 2016-03-19

**Authors:** Elias de França, Ana Paula Xavier, Vinicius Barroso Hirota, Sônia Cavalcanti Côrrea, Érico Chagas Caperuto

**Affiliations:** a Laboratory of Exercise and Movement Sciences, Mackenzie Presbiterian University, São Paulo, SP, Brazil; b Laboratory of Human Movement, Department of Physical Education, São Judas Tadeu University, São Paulo, SP, Brazil

**Keywords:** Pace strategy, High-intensity intermittent run test, Central and peripheral fatigue assessment

## Abstract

This data paper contains data related to a reproducibility test for running pacing strategy in an intermittent running test until exhaustion. Ten participants underwent a crossover study (test and retest) with an intermittent running test. The test was composed of three-minute sets (at 1 km/h above Onset Blood Lactate Accumulation) until volitional exhaustion. To assess pace strategy change, in the first test participants chose the rest time interval (RTI) between sets (ranging from 30 to 60 s) and in the second test the maximum RTI values were either the RTI chosen in the first test (maximum RTI value), or less if desired. To verify the reproducibility of the test, rating perceived exertion (RPE), heart rate (HR) and blood plasma lactate concentration ([La]p) were collected at rest, immediately after each set and at the end of the tests. As results, RTI, RPE, HR, [La]p and time to exhaustion were not statistically different (*p*>0.05) between test and retest, as well as they demonstrated good intraclass correlation.

**Specifications Table**

**Table:** 

Subject area	*Sport Science,*
More specific subject area	*Psychology of Sport, Physiology of Sport*
Type of data	*Table, text file*
How data was acquired	*Borg scale (6–20), Polar S810i, YSI 1500 Sport Lactate Analyzer ™*
Data format	*Analyzed*
Experimental factors	*Hemolyzed lactate concentration was determined enzymatically in whole blood, using a YSI 1500 lactate analyzer (YSI Inc., Yellow Springs, Ohio, USA).*
Experimental features	*Blood lactate was extracted from the ring finger of the participants’ right hand.*
Data source location	*São Paulo, Brazil*
Data accessibility	*Data is supplied in this article*

**Value of the data**

● To assess running pace strategy change.
● To assess changes in recovery time length, between exercise bouts.
● To assess time to exhaustion in an intermittent exercise.
● This protocol might be used to assess performance status changes.

## 1 Data

Reproducibility data of a high-intensity intermittent running test to assess changes in running pace strategy concomitant to central and peripheral variables related to fatigue onset are presented in Supplementary Table 1 and 2. According to the ANOVA two-way test (test *versus* exercise bouts), at rest and in all exercise bouts there was no significant interaction time between the two tests for the variables analyzed, assuring the test reproducibility (see Supplementary Table 1B for HR, Supplementary Table 1C for [La]p, and Supplementary Table 1D for RPE).

The laboratory running test showed to be severe enough to cause fatigue and to promote disengagement of the activity with high mental and metabolic stress [1], so the participants were able to perform ~three exercise bouts (i.e., ~9 min, not counting the RTI between exercise bouts) before entering volitional exhaustion.

## 2 Experimental design, materials and methods

### 2.1 Participants

We tested ten (*n*=10) healthy, male, university students, aged 24±4 years, weight 76.5±6.4 kg, height 178.3±6.0 cm, body fat: 9±2% (assessed by a Lange skinfold calipers, according to Jackson and Pollock) [2], VO_2_ max 52.93±3.05 ml Kg^−1^ min^−1^, Onset Blood Lactate Accumulation (OBLA) 3.38±0.56 mmol/L. The participants did not use any ergogenic supplements in the last 6 months preceding the test. All participants were amateur athletes, and all of them were used to running on treadmills.

Participants were instructed to refrain, at least 48 h from intense physical activity before the test; they were also instructed not to use any kind of food or substances that might interfere with the performance in the central nervous system or change physiological or biochemical parameters, such as alcohol or caffeine for at least 24 h before the test. In addition, 12 h before the test we provided the participants with a printed material that contained a standard high-carbohydrate (~70% kcal), low fiber, and fat (~15% kcal) diet that should be repeated on the crossover trial. It is important to mention that was not monitored whether participants strictly followed our orientations.

### 2.2 Ethics

All procedures were approved by Mackenzie Presbiterian University ethical committee under the protocol number (CEP/UPM n°1440/04/2012). Participants signed an informed consent form and were alerted, even that, they were free to abandon the study if desired any time.

### 2.3 Experimental design

The experiments were performed in a randomized and crossover way where the participants functioned as controls of themselves. They came to the laboratory in three different occasions, always in the afternoon, at the same time of the day and the same day of the week (every Monday around 4:00 p.m.). Room temperature (°C) observed was around 23±3 and humidity (%) was of 70±5.

The first visit was to present the study design and to sign the informed consent form, followed by a first evaluation (anthropometric assessment; OBLA [3] and VO_2_ max estimation) for the individualized prescription of the physical test intensity, in a motorized treadmill (Aegean 6200). In the second and the third visit to the laboratory, the tests to verify the reproducibility were carried out.

### 2.4 Determination of OBLA and VO_2_ max estimation

This protocol is adapted from Denadai et al. [3] The participants completed a standard incremental treadmill test until volitional exhaustion (Aegean 6200, Porto Alegre, Brazil). The initial running speed was 10 km h^−1^, with the treadmill grade set at 1%. Participants completed submaximal stages of 3-min, with 1 km h^−1^ increases between stages. At the end of each stage, participants jumped off to the sides of the treadmill belt. A right-hand finger capillary blood sample (100 µl) was taken within 20–30 s, after which participants resumed running. Blood lactate concentration was analyzed by an automated analyzer (YSI 1500 Sport Lactate Analyzer ™). Heart rate was recorded during the whole test (Polar S810i). OBLA was determined according to Cheng et al. [4]. VO_2_max was determined according to ACSM [5] metabolic equation for estimating gross VO_2_ at running.

### 2.5 Laboratory running test

The test was performed in the same motorized treadmill (with 1% incline grade) where we analyzed the OBLA. The protocol consisted in a 9 min incremental warm up (e.g., a participant who had a OBLA speed at 15 km/h; began the warm up with three minutes at 12 km/h, followed by three minutes at 13 km/h and then three minutes at 14 km/h) followed by a 1-min walk interval. After the warm up, participants performed an intermittent run until volitional exhaustion, with sets of 3-minutes exercise bouts, interspersed with passive RTI determined by the participants in the first test (detailed description of the rest interval determination below). To induce a higher rate of metabolic and mental stress during the test [1] the intensity of the run was determined (by interpolation) at 1 km/h above the OBLA speed. (The mean ±SD velocity (15.19±0.81 km h^−1^) during the exercise bouts was a little higher than critical velocity [6]).

During all tests, the participants were encouraged by strong verbal stimulation to perform their maximum effort and they were informed of the exercise bout remaining time every 30 s.

### 2.6 The rest time interval

This variable has been created to test the possible changes in pace strategy, which might influence recovery between exercise bouts. For this, before the first test, participants were informed that in the first test they could choose the RTI between exercises bouts on the treadmill: the minimum RTI would be 30 s and the maximum would 60 s. Usually 60 s of RTI is sufficient to promote an increase of 100% in the total running distance when compared to continuous running (when exercise is performed at the similar intensity used in our protocol) [6]. This occurs because RTI between high-intensity physical exercise series prevents RPE from reaching critical values early, i.e., restores the ability of the Central Nervous System (CNS) to stimulate muscle work, thus keeps the athlete engaged in the exercise [7], [8], [9].

It has also been settled that the RTI values of the first test would be the maximum RTI of the next test, for example, if in the first test the RTI was 40 s between the first and second exercise bouts, and 50 s between the second and third exercise bouts, these same RTI values would be the maximum amount of RTI in the next test in their respective moments, (we believe that RTI control strategy ensured the test reproducibility). An important point is that the participants could rest less time if they feel like to resume the test and perform another set of 3 min of exercise (this idea was created to test the effectiveness of possible ergogenic interventions on RTI and consequently pace strategy). When the participants felt that they could not perform, or were not willing to try to perform another set of 3 min, the test was given as completed.

### 2.7 Materials and data collection procedures

Data collection was performed before the start of the test (*i.e*., at rest) immediately after each exercise bout and in the end. At all times, before the volunteers begin the test, and immediately after each exercise bout, we measured RPE (assessed by Borg scale [10]), collected the Heart Rate (HR) value (with a Polar S810i series heart rate monitor, with HR measurement capacity set at recording at every 5 s) and [La]p (through the YSI 1500 Sport Lactate Analyzer ™; device with an error up to 0.10±5 mmol/L).

The collection of blood samples for [La]p analysis were obtained by collecting around 100 µl of blood from the ring finger of the participants׳ right hand (for that we used an automatic Softclix II AccuCheck piercingdevice from Roche and the blood was collected to a heparin capillary tube), then it was injected into the lactate analyzer device using a 50 µl pipette.

The same three researchers were responsible for collecting all data from the exercise bouts, one for blood collection, one for collection of HR and the third for RPE report according to the Borg scale.

### 2.8 Statistical analysis

The results were presented as mean (for parametric data), ±Standard Deviation and median (for non-parametric data). After the assessment of the normality of the data (with Shapiro-Wilk test) we used the paired Student *t*-test (on Rest Time Interval, HR, [La]p and RPE) and Wilcoxon signed-rank test (on TTE) to verify the mean differences between test and retest. In addition, we evaluated the magnitude difference (Effect Size-ES) between the two tests in the variables by Cohen׳s *d* (for parametric data) and by Cliff׳s Delta (for non-parametric data) [11]. We also evaluated the Confidence Interval (CI) set at 95%.

We used the ANOVA two-way test (tests x exercise bouts) to evaluate interaction time and time effect between HR, RPE and [La]p.

All statistical tests were conducted using the Statistical Package for the Social Sciences 20 (SPSS-20). Significance was set at *p*<0.05.
